# Immunity-Based Optimal Estimation Approach for a New Real Time Group Elevator Dynamic Control Application for Energy and Time Saving

**DOI:** 10.1155/2013/805343

**Published:** 2013-07-09

**Authors:** Mehmet Baygin, Mehmet Karakose

**Affiliations:** ^1^Computer Engineering Department, Ardahan University, Ardahan, Turkey; ^2^Computer Engineering Department, Firat University, Elazig, Turkey

## Abstract

Nowadays, the increasing use of group elevator control systems owing to increasing building heights makes the development of high-performance algorithms necessary in terms of time and energy saving. Although there are many studies in the literature about this topic, they are still not effective enough because they are not able to evaluate all features of system. In this paper, a new approach of immune system-based optimal estimate is studied for dynamic control of group elevator systems. The method is mainly based on estimation of optimal way by optimizing all calls with genetic, immune system and DNA computing algorithms, and it is evaluated with a fuzzy system. The system has a dynamic feature in terms of the situation of calls and the option of the most appropriate algorithm, and it also adaptively works in terms of parameters such as the number of floors and cabins. This new approach which provides both time and energy saving was carried out in real time. The experimental results comparatively demonstrate the effects of method. With dynamic and adaptive control approach in this study carried out, a significant progress on group elevator control systems has been achieved in terms of time and energy efficiency according to traditional methods.

## 1. Introduction

The scheduling of the group elevator systems, which has been used for vertical transportation at high-rise buildings, has an important place in our daily lives. Aiming to give rapid service to the crowd population in the building, such systems have very complex structure. One of the issues that people usually complain inside building is vertical transportation service of the building [1]. The studies on this topic continue for a long time, and important developments are provided.

The group elevator systems are structures, which elevate the population in the building from one floor to another by using more than one cabin in the widest sense. Generally managed from one control center in a coordinated manner, such systems are finding optimal ways according to the condition of calls, and they apply such ways on cabins. However, the width of the optimal way space makes this problem hard to solve in the best way. Because there are the option of *n* ^*p*^ different ways in a building having *n* storey and *p* cabin [2, 3]. Actually, what is expected from the group elevator systems is the average performance of such cabins instead of individual performance of elevator. In addition, the basic criteria at such performances are to minimize the average waiting time of passengers and energy consumption values of cabins [3, 4]. Generally, by considering such parameters, the most optimum way is selected from the way space, and these are applied to the system. Actually, there are many factors, which affect average waiting time; these can be counted as lack of some information like number of passengers, elevator speed, and so on. For instance, the passengers have to wait new coming elevator for a long time because they missed the elevator a few minutes ago. In addition to this case, although no one uses elevator because of call of any empty floor, the passengers often wait for closing the doors [5]. All of this and similar circumstances are caused by lack of traffic information, and those waiting directly affect the average waiting time. However, despite all such circumstances, average waiting time can be approximately calculated as shown in the following equation [6, 7]:

(1) AWT=0.4 INT, for  car  loads<50%,AWT=[0.4+1.8 PCC−0.772]INT, for  car  loads>50%,

where AWT, average waiting time, *P*, numbers of passenger, INT, interval (the main floor average time), and CC, car capacity.

For the most suitable car allocation at the past, although some mathematical approaches are thought, today soft computing techniques and artificial intelligence algorithms are often applied to this kind of system. In addition, such calculations can be made in shortest time with advanced computer systems, and the most optimum car can be allocated to the users [3].

One other criterion of the group elevator systems is energy consumption values in cabins. Although the energy consumption of the elevator occurs at the moment of stop start, it consists of two sections as travel and standby position. The consumption during travel (stop-travel-start) compromises more than 70% of total energy consumption [8]. The most important problem here is irregular energy consumption of cabins. For example, working of elevators without any pattern causes some cabins to operate more and to wear off. This parameter is considered in applications to be made for the removal of this condition, and load distribution of cabins is attempted to be equalized in parallel with average waiting time. Generally, it is very hard to determine daily energy consumption of elevator. However, Schroeder makes many measurements in many elevators and creates a formula to calculate this value. In (2), according to the Schroeder method, daily energy consumption amount of an elevator has been given. In this equation, TP shows general course time, and this term varies depending on drive mechanism type and speed of elevator [7]. The strongest point in the Schroeder method is to determine the ST value. Because the incoming calls are not known earlier, accordingly, how much the lifts operate during day is not predetermined. For this reason, this value is determined by either making certain measurements or by using certain approaches [7, 9]. Because the energy consumption of elevators is mostly caused by stop-start movements, this value directly affects the equation [9]

(2) $$\begin{array}{c} \text{EC}=N*\frac{R*\text{ST}*\text{TP}}{3600}, \end{array}$$

where EC is energy consumption (kWh/day), *N* is cabin number, *R* is motor ratings (kW), ST is daily start number, and TP = time factor.

The most important factor affecting both the average waiting time of passengers and energy consumption in group elevator systems is traffic time of buildings [2, 9]. This is very important issue because passengers use elevators much in certain hours, and they rarely use cabins in rest of their times. In the observations, 4 different traffic times are determined in any building, and such traffic hours and passenger density created depending on those hours are considered in either real world or computer simulations. Depending on those hours, the first of created density is uppeak, and it includes the hours when people come to work at mornings at the office building [1, 4, 6, 8]. The calls made in traffic are generally upward position, and these upward calls reach peak points at mornings. The second traffic movement is downpeak. This situation includes arrival time from work, and calls from floors are generally downward [5, 9]. Other traffic time is seen at lunch times. It contains the time frame at which building population is going out at lunch break and is coming back to work [10]. Traffic condition is random situation between floors. This traffic condition is caused by passing of people inside building between floors, and it is traffic condition, which is seen during working hours [3, 8].

**Figure 1:**
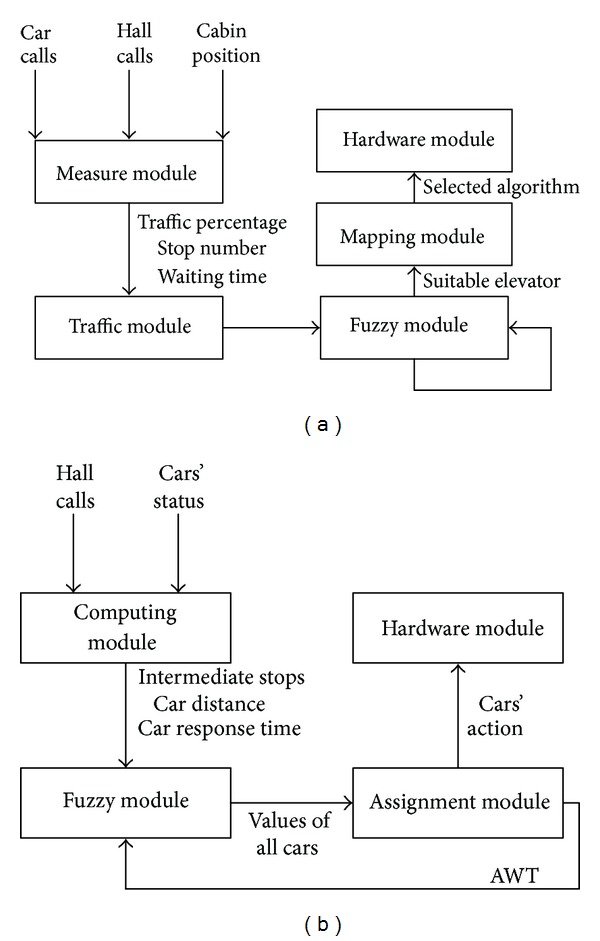
The closest two studies in literature (a) [2] (b) [4].

There are various studies made in literature on group elevator systems. Either reduction of average waiting time of passengers or reduction of energy consumption is wide in literature. In such studies on which certain traffic conditions are considered, the soft computing techniques like genetic algorithms, fuzzy logic, particle swarm optimization, and artificial intelligence [4, 6, 8]. However, sheer number of parameters in elevator systems, width of solution space, and failure to determine incoming calls earlier make scheduling problem hard to solve [2, 3]. Despite these lacks, an important development has been performed from the first day of appearance of group elevator systems, and still continues to develop. Some studies, which have been made on literature within scope of working has been examined, such studies have been classified as aim, used method, traffic condition, number of elevator, and simulation forms. However, some lacks have been found in studies performed in literature in this classification. This study aims at elimination of lacks in literature with this study, and brings a new point of view to the solution of scheduling problems for group elevator systems. Two examples have been given to the studies which have been made in the literature Figure 1.

As seen in Figure 1, the procedures in the studies made in literature are performed over one algorithm with certain number of cabins. These limitations bring many problems. For example, subjecting to only one algorithm does not guarantee that the application always gives best results. Because each algorithm has different ways to reach better, and for this reason, the best results, which have been found by each algorithm, are different from each other. In this kind of system, it is evident that better results can be obtained by using different algorithms together. One other lack in the studies made on group elevator systems is limited number of cabins. This situation is directly associated with use of only one algorithm in the system. The largest lack here is that the algorithm selected gives better results in the designated number of algorithm. However, the results are reverse from one expected when you exclude the number of cabins. This study makes clear all this and similar conditions, and the studies made on literature gains new dimension. The artificial immune systems, genetic algorithms, and DNA computing algorithm have been used in the study performed for reduction of average waiting time of passengers and providing energy efficiency in group elevator systems. In addition to these methods, an estimation algorithm has been developed, the relation of such methods has been provided with fuzzy logic, and the most suitable algorithm has been aimed according to the calls and cabin conditions of the system in adaptive way. The results which have been obtained from fuzzy logic are sent to the microprocessor-based experiment set, and the performance and accuracy of the system have been observed. The system procedure and methods used for control algorithm, which is recommended in 2nd section within scope of this study, have been examined; the simulation results and test results which have been obtained from these algorithms in 3rd section have been given. The obtained results are assessed in 4th and final section of the study.

## 2. Proposed Approach

The group elevator systems have a very complex structure. The sheer number of input parameters and many options as output makes these systems hard to solve. Especially the conditions as unknown building population or unknown floor by a passenger are basic factors in creation of wide solution space. However the developed electronic and computer world is useful for similar problems and used to solve this. Especially, making computational procedures about milliseconds makes detection of most suitable option from wide solution space.

There are some basic principles required to perform by system while group elevator systems are in service. These principles, which have been given in list below, are considered in this study.

- The elevator cannot pass up cabin call. It must complete these until all calls in present direction, and the cabin calls in reverse direction must be ignored in this process. After present direction of elevator returns reverse, the rest of calls must be completed.
- If there is enough space inside cabin before change of direction of system, it can collect floor calls in same direction.
- If there is no one at the point where floor call is made, this floor call can be ignored.
- It is required to do less stop-start movement as much as possible to provide energy efficiency of system. To perform this, no more than one cabin goes to the same floor call.
- It is required to do feasibility study before for population inside building and frequency to use elevator by this population in order to increase efficiency of methods to be used in the system.

**Figure 2:**
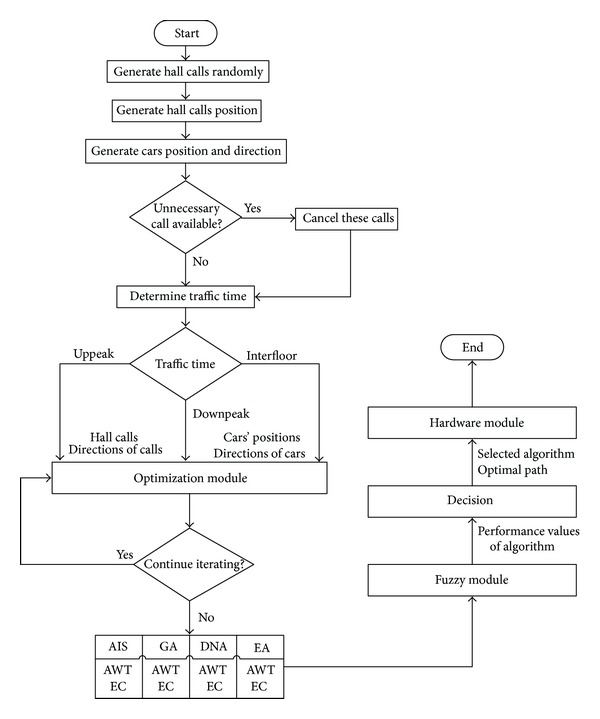
Flow chart of the proposed approach.

This study aims at reduction of average waiting time of passengers and providing energy efficiency. The proposed control approach primarily consists of 3 main modules. The first module is optimization module, and calls are evaluated here according to the 4 different optimization methods. The results which have been obtained from the assessment made according to the average waiting time and energy efficiency are sent to the 2nd module, the fuzzy module. In this module, the method to be applied on call pool is determined, and optimum way must be given as exit. The optimal way obtained from the fuzzy module is sent to the hardware module and is tested on experiment set. A flow diagram is given in Figure 2, which summarizes the system.

### 2.1. Optimization Module

A group elevator system is modeled with modules designated within scope of this study. The optimization methods, which are often used in wide problems where solution space is wide, are used within scope of study. Three different optimization methods and 1 estimation algorithm are used in this performed optimization module. As optimization methods, clonal selection algorithm, genetic algorithms, and DNA computing algorithm are used from artificial immune system.

#### 2.1.1. Clonal Selection Algorithm

The artificial immune algorithms which are proposed by taking human immune system as example incorporate negative and clonal selection algorithms. While negative selection algorithm is used in determination of undesired situations, the clonal selection algorithms are often used in optimization and pattern recognition problems [11, 12]. The pseudocode of the clonal selection algorithm, which has been used for optimization purpose in this study, has been given below.

**Step 1**

The antibodies compromising body antibody repertoire constitute the initial solution set.

**Step 2**

Degree of similarity of antibodies is calculated.

**Step 3**

*n* pieces of highest similar antibody are selected.

**Step 4**

Proportional to the degree of similarity of the *n* pieces of the selected antibody, the high degree of similarity cloning of antibody is carried out.

**Step 5**

Antibodies with a high degree of similarity are exposed to the mutation as a manner to become less.

**Step 6**

The degrees of similarity of mutated clones are determined.

**Step 7**

*n* pieces of highest similar antibody are selected again.

**Step 8**

Change of *d* pieces of antibodies in lowest degree of similarity with newly produced antibodies is realized.

#### 2.1.2. Genetic Algorithm

Genetic algorithms are search and optimization methods are aiming to find the best result from the problem space in the widest sense [13, 14]. Offering extremely fast and efficient solutions, this method has been used in this study as one of the optimization methods, which have been used during simulation. A pseudocode, which summarizes the operating principle of genetic algorithms, is given below.

**Step 1**

Initial population of randomly generated sequences of binary numbers.

**Step 2**

A certain amount of element is selected for the solution.

**Step 3**

Crossing is applied to new population.

**Step 4**

Mutation process is applied for the same population.

**Step 5**

Affinity values of elements of these populations are found.

**Step 6**

Step 2 is repeated until it reaches the maximum number of transactions.

#### 2.1.3. DNA Computing Algorithm

DNA computing algorithm is a method, which is mainly used in literature, and it is soft computing technique used in solution of the NP hard problems [15, 16]. The operating principle of this method used in this study as assessment criteria is similar to the genetic algorithms, and the pseudocode which summarizes this algorithm is given below.

**Step 1**

First population is created.

**Step 2**

DNA sequences are converted to numeric values.

**Step 3**

Affinity value is calculated for each element.

**Step 4**

Crossing process is applied to individuals in the population and the new population is obtained.

**Step 5**

Mutation of the enzyme is applied to new population.

**Step 6**

Mutation of the virus instead of the deleted elements is applied.

**Step 7**

Affinity value of population is determined. If newly found population value is better than original value, it is changed and it is continued from 3rd step until maximum procedure number is reached.

#### 2.1.4. Proposed Estimation Algorithm

**Figure 3:**
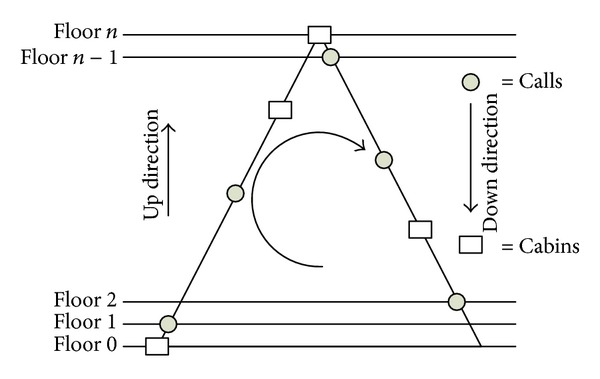
Proposed estimation algorithm.

Within the scope of the study, an estimation algorithm has been proposed in order to bring a different point of view to group elevator systems. Together with this algorithm, it has been aimed to enable the energy efficiency of the cabins. Since the results obtained by optimization methods used bring overload to some cabins, it causes some cars work more and some cars work less. This is an undesired situation and it both directly reflects to average waiting time and energy consumption values of the cabins. In principle, it is expected from an elevator system that energy consumption values should resemble each other so that all the cabins can work efficiently to decrease waiting time of the passengers. For this reason, a diagram, which summarizes the estimation algorithm performed, has been given in Figure 3.

Proposed estimation algorithm considers the longest route that the elevators can follow. As it is shown in Figure 3, left side of the triangle shows upper direction, and right side of the triangle shows the down direction. The rectangles present in these sides show the instant position of the cabins and rounds show the calls made. The longest route that the elevators can follow is to go to the top floor and turn back to the ground floor, and this situation has been shown with an arrow in the triangle. In other words, the cabins in the triangle move clockwise and calls settle into the edges of the triangle according to the directions. Proposed estimation algorithm is based on answering calls available to the current direction of movement of the cabins. Pseudocode, which summarizes working principle of this algorithm, has been given below.

**Step 1**

Determine number of user floor, number of cabins, position of cabins, and their directions.

**Step 2**

Randomly directed calls are created.

**Step 3**

Calls at ground floor are always arranged upwards and calls at top floor of building are arranged downwards.

**Step 4**

Cabins and randomly produced calls are placed on triangle according to directions.

**Step 5**

The arrival time to calls is calculated according to affinity function of cabins.

**Step 6**

Calls having low arrival time are shared to proper cabins and these calls are deleted from call pools.

**Step 7**

If there are remaining calls, proceed from the 5th step.

**Step 8**

Proceed from Step 2 until maximum iteration number is reached.

### 2.2. Fuzzy Module

**Figure 4:**
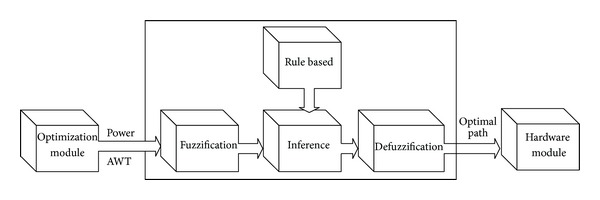
Fuzzy logic module.

Another part in proposed control algorithm for group elevator systems is fuzzy module. The diagram which summarizes fuzzy systems consisting of generally four fundamental parts has been given in Figure 4 [17, 18].

**Figure 5:**
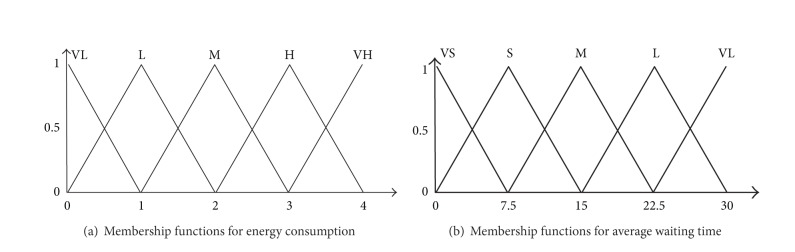
Input membership functions.

Fuzzy module in the system takes energy consumption values and average waiting time coming from optimization module and sends to fuzzy part. In step of fuzzification, there is energy membership input function and average waiting time membership input function. The input functions have been given in Figure 5.

**Figure 6:**
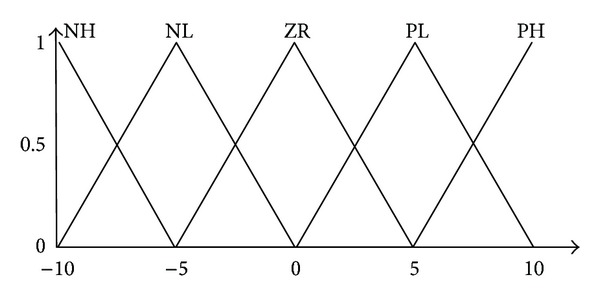
Output membership functions.

The values obtained from membership input function are sent to inference part to be evaluated. Here, Mamdani method is used and the system has defined 25 different rules for this procedure. The values obtained from inference part are sent to clarification part to be converted into reel values. In this part, optimal route is defined according to exit membership functions defined. In Table 1 given below, rule base used in fuzzy module has been given and membership exit function has been shown in Figure 6.

### 2.3. Hardware Module

**Figure 7:**
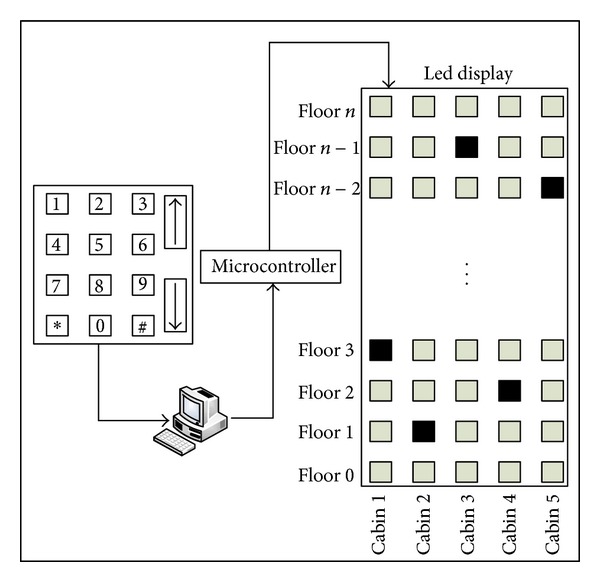
Hardware module.

The final part of the practice performed within the scope of the study is hardware part. In this part, optimal path coming from fuzzy logic module has been evaluated in the microprocessor-based experiment set and the accuracy of the system is tested. Also, led mechanism connected to experiment set represents a building and the cabins in the building. The practice made with this module has been brought into practicable condition by taking from being simulation. A diagram, which summarizes a hardware module used in the system, is as given in Figure 7.

## 3. Experimental Results

Within the scope of the study done, it has been aimed to decrease the average waiting time of the passengers and enable energy efficiency with proposed control approach. In direction of these purposes, the application performed has been performed by using 3 modules and the results are perceptibly observed. Simulation stage of the application has been prepared in a computer environment with Windows 7 operating system including 2,53 GHZ speed, 4 GB RAM, Intel Core i5. The study done has been performed in Matlab R2009b platform. An experiment set has been used for hardware stage of the application. This experiment set has been worked in 20 MHZ speed, and RS-232 serial communication line in 9600 bandwidth has been used for communication. The results coming from simulation stage are transmitted to microprocessor-based experiment set through serial communication line. In addition to this, in order to be able to define the starting status of the cabins and let them make call after the system starts, a numeric keypad is used. Building and cabin specifications used as base at simulation stage are as given in Tables 2 and 3.

**Figure 8:**
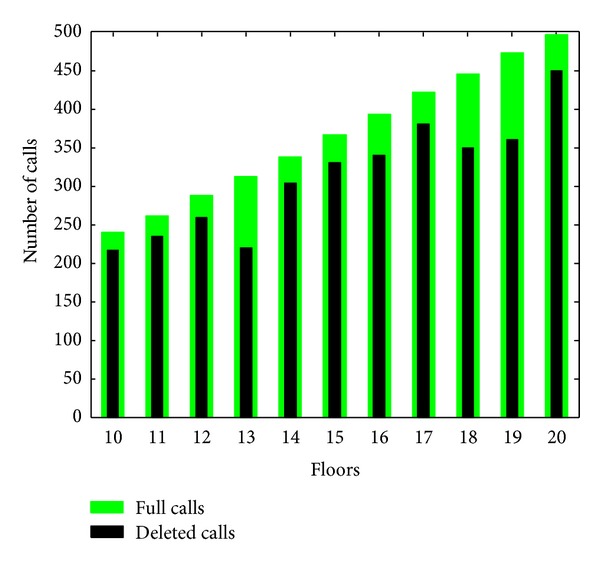
Calls according to number of floors.

With the application performed within the scope of the study, total average waiting times and energy efficiencies of the cabins have been calculated. Random 500 numbers of calls have been created within the frame of the application, and approximately 80% of these calls have been arranged as to be downside and upside to be able to obtain up and down traffic condition. For traffic condition in inter floors, half of 500 numbers of calls have been arranged so as to be downward and the other half has been arranged so as to be upward. The distribution of the calls according to the floor has been shown in Figure 8.

Depending on the number of floors, the number of the calls increases. In the study done, if there is nobody in the floor to be made, the cabins are enabled to pass over this call. In order to perform this purpose, 20% of the current calls have been cancelled, computational procedures are started after this point. The cancellation of invalid calls provides an important advantage regarding energy efficiency and average waiting time. In Table 4, average waiting time changing to the floor situations and algorithms have been shown.

**Figure 9:**
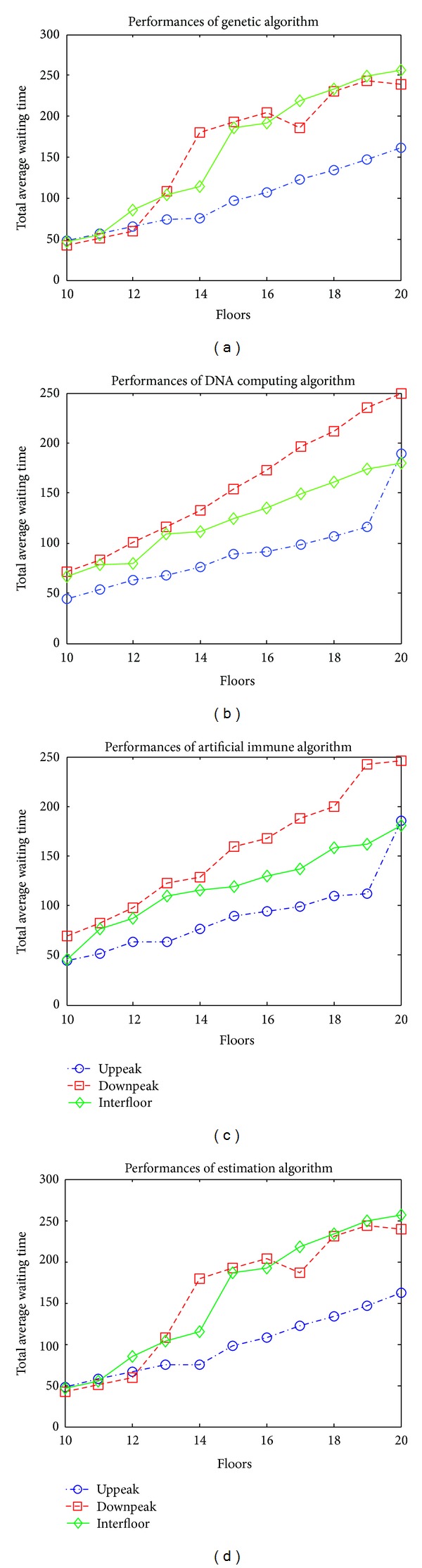
Total average waiting time performances of algorithms.

The performance of algorithms according to the number of traffic peak hours and floors is shown in Figure 9. In the application performed, 3 different traffic times, genetic algorithm, DNA computing algorithm, artificial immune system algorithm, and estimation algorithm have, respectively, been applied. At practice stage, total 11 different floor conditions have been considered, and algorithms through 2, 3, 4, and 5 cabins for each floor have been applied. Average waiting times obtained for each cabin and floor have been added separately and divided into total cabin number. When Table 4 is examined, it has been seen that waiting time increases in parallel with the floor number. Furthermore, the performances that algorithms have shown under different traffic conditions are different from each other. For example, while artificial immune system algorithm gives the best result in uppeak traffic condition, estimation algorithm in downpeak traffic algorithm shows the best performance. There are so many factors which affect average waiting times in group elevator systems. As the complete of factors such as number of passengers or hall calls are not known, it cannot completely be calculated regarding which algorithm that provides the best solution. With this study done, a system with adaptive structure has been created and significant results have been obtained from the point of waiting time and energy efficiency.

**Figure 10:**
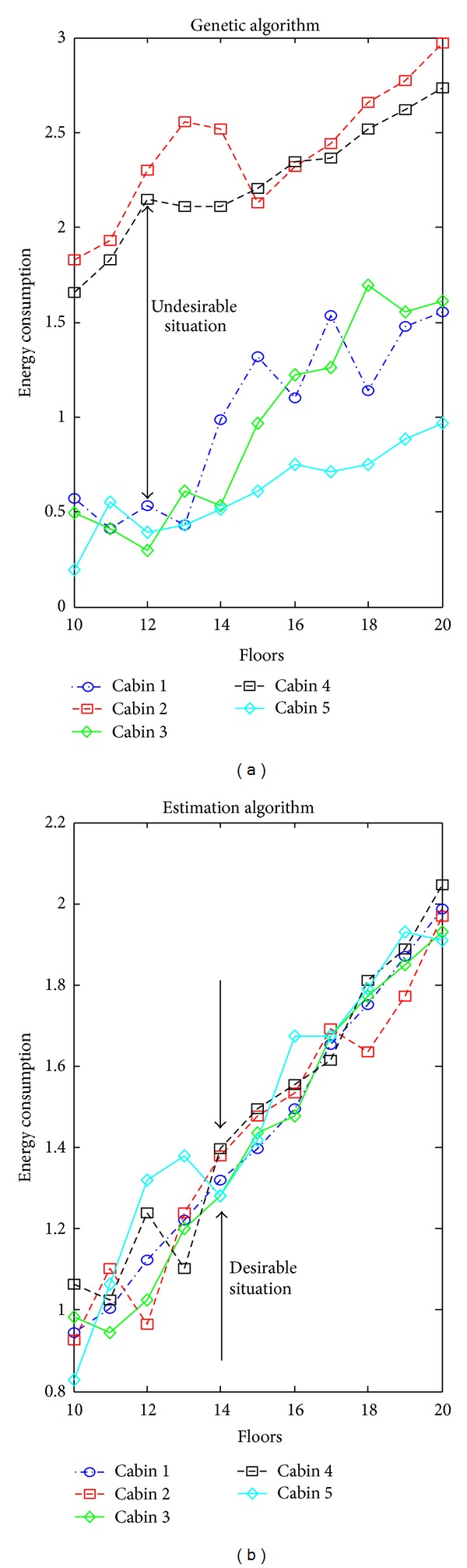
Energy consumption of cabins according to floors.

Another parameter within the scope of the study done is the energy efficiency of the cabins. One of the most important disadvantages of the group elevator systems is the lowness in energy performances arising from working principles. 70% of the energy that elevator systems consumed occurs while the cabins stop and start. Furthermore, this stop and start movements are seen more frequent in some cabins compared to the others. This situation causes some cabins to consume more energy and wear down. In principle, it is expected from group elevator systems that total energy to be consumed is provided to be stably distributed to the cabins according to the stop-and-start number. Because of working principles of optimization methods, some cabins are overloaded. With the study performed, this situation is aimed to be removed and the energy is provided to be distributed as efficiently. An example of the energy distributions that the cabins consumed has been shown in Figure 10 according to the traffic and algorithm conditions.

As it can be understood from the figure, there is an unbalanced load distribution between the cabins according to the optimization methods. With the proposed estimation algorithm, this situation is prevented and it has been enabled to make the cabins share the calls equally. The equal distribution of the calls to all the cabins provides a significant efficiency from the point of energy. By this means, a development occurs in the usage times of the cabins and the wearing times of the cabins extremely decrease.

It is expected from group elevator systems that average waiting time of the system and energy efficiency should be provided at the same time. Because of the fact that in some circumstances, while average waiting time is good, energy efficiency shows inverse proportion to this time. To prevent this situation, a fuzzy system has been designed. The fuzzy system designed takes energy values and average waiting times coming from 4 different algorithms to the traffic conditions as input parameter. Then, it evaluates these parameters and provides the most optimal path to be determined. The basic purpose in fuzzy system designed is to find the lowest average waiting time and the best result of energy efficiency. In Table 5, the values rising from fuzzy logic and the methods to be applied according to these values have been shown.

**Figure 11:**
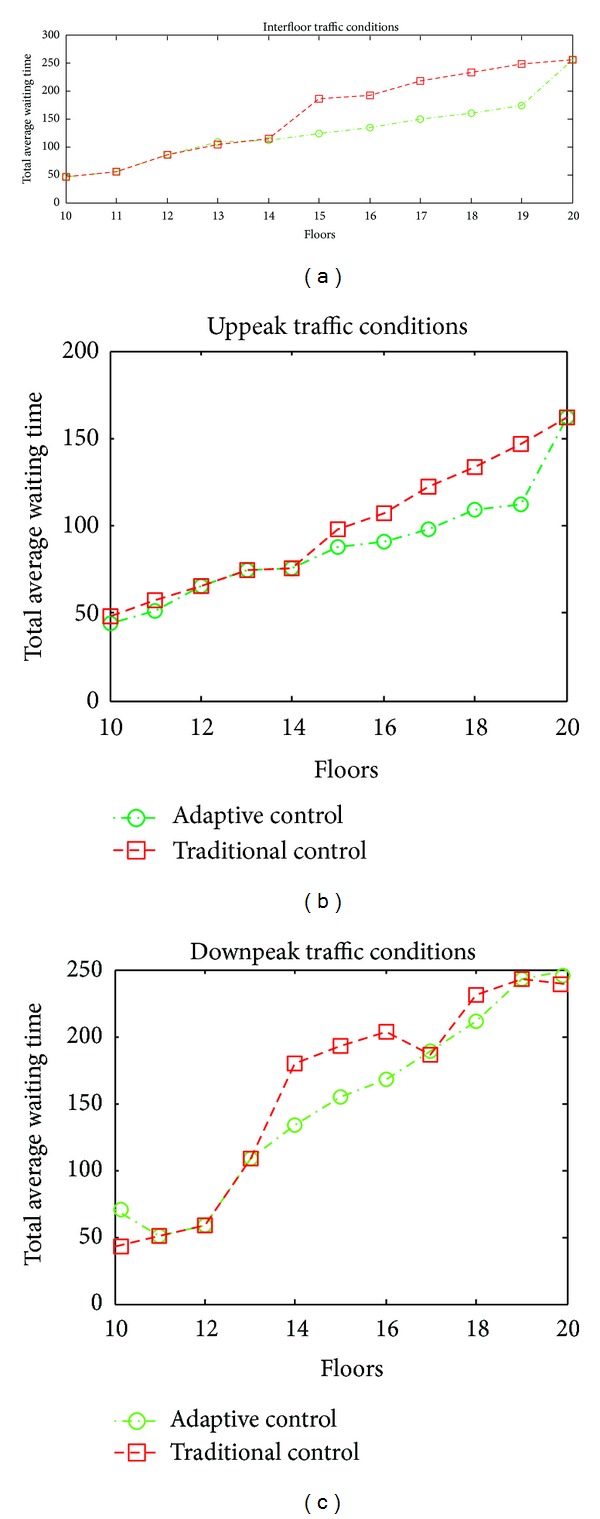
Comparative results for classical methods and the proposed approach.

Thanks to this performed study, an adaptive approach is developed related to group elevator control systems. It is aimed at making this approach reduces the average waiting time of passengers and provides energy efficiency. The proposed adaptive approach and four different algorithms are combined and fuzzy logic module of the system and the results from these different algorithms are provided to evaluate. In Figure 11, traditional methods on group elevator control systems and changing total average waiting times depending on number of floors of proposed adaptive approach.

**Figure 12:**
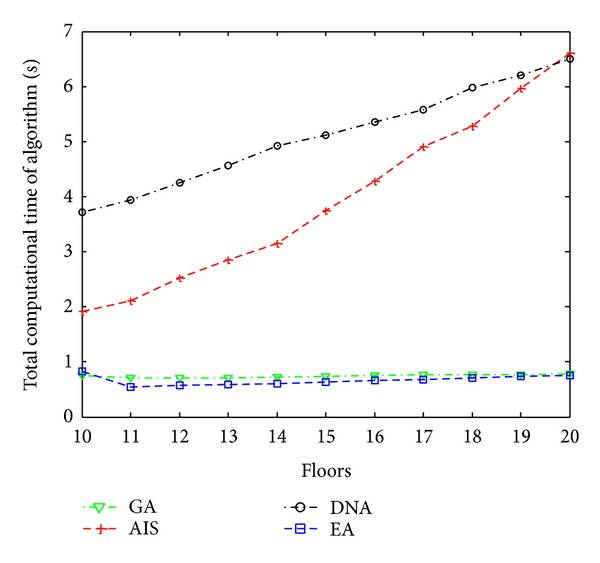
Total computational time of algorithms.

These findings obtained in simulation platform are sent to the experiment set and the accuracy of the system has been tested.  Figure 12 shows the computing time passing from simulation platform to the experiment set according to the floor number and traffic condition for each algorithm separately.

## 4. Conclusions

The elevator systems have a structure that constantly renews itself since day one of the emergence. Today, the increase of high-rise buildings is the biggest factor for this situation. Group elevator control systems which were developed in order to provide faster service to resident are computerized to provide both time saving and energy efficiency. Group elevator control systems which have very large place in the literature are not incomplete in terms of efficiency because all of parameters are not taken into consideration.

In this study, an immune system-based approach for the control of group elevator control systems is conducted, and reducing of the average waiting times of passengers and providing of energy efficiency of system are aimed to provide. In the scope of this study, the adaptability of this application to all building structure is aimed to provide by creating building models from 10 floors to 20 floors and from 2 cabins to 5 cabins. In the next step, calls randomly generated according to traffic hours are sent to optimization module which includes immune system algorithm, genetic algorithm, and DNA computing algorithm to determine average waiting times and energy consumption values of cabins. The average waiting time and cabin energy consumption values that they are acquired from optimization module due to each algorithm are sent to fuzzy system module which is designed in the scope of the study to determine system performance. The optimum way for formed building models and traffic hours are aimed to determine by providing obtained values of fuzzy system. Finally, this optimum way obtained from fuzzy module is tested with hardware module.

This adaptive and dynamic control approach for elevator systems provides efficiency approximately between 5% and 20% on average waiting times of passengers in comparison with traditional methods. In addition to this, a significant gain was achieved related to energy efficiency which is another expected parameter in elevator systems, provided that calls to cabins are as evenly distributed as possible. Future studies target to further increase the efficiency of the system and perform these applications which are performed on a real elevator system.

## Figures and Tables

**Table 1:** Rule base of fuzzy logic module.

Input	Energy consumption				
	VL	L	M	H	VH
Average waiting time					
VS	NH	NH	NL	NL	ZR
S	NH	NL	NL	ZR	PL
M	NL	NL	ZR	PL	PL
L	NL	ZR	PL	PL	PH
VL	ZR	PL	PL	PH	PH

**Table 2:** Building parameters.

Number of elevators	From 10 to 20
Number of cabins	From 2 to 5
Floor height	3,5 m
Building type	Office
Building population	500
Building occupancy	80%

**Table 3:** Cabin parameters.

Cabin rate	13 person
Velocity	1,6 m/sn
Door open	3 sn
Door close	4 sn
Passenger transfer time	1,2 sn
One floor passing time	2,2 sn

**Table 4:** Average waiting time of passengers (s).

Floor	GA			DNA			AIS			EA		
	Uppeak	Downpeak	Inter floor	Uppeak	Downpeak	Inter floor	Uppeak	Downpeak	Inter floor	Uppeak	Downpeak	Inter floor
10	52,1	75,1	68,7	**44,8**	71,5	66,2	44,4	69,9	**45,7**	48,5	**42,7**	46,8
11	59,2	87,7	79,7	53,3	82,7	78,3	**51,2**	82,2	76,3	57,2	**50,4**	**55,4**
12	**62,3**	101,5	91	63,5	100,6	**79,6**	62,8	97,5	86,5	65,9	**59,2**	85,5
13	72,9	117,2	**103,7**	67,6	**115,7**	108,5	**63,6**	122,8	109,2	74,5	108,3	104,1
14	78,3	135,9	**110,9**	75,9	133,1	111,4	76,4	**128,8**	115,4	**75,4**	179,7	114,3
15	**88,3**	159,9	127,6	89,4	**154,1**	124,7	89,2	159	**119**	97,6	192,6	186,6
16	93,8	174,6	137,9	**91,1**	172,8	134,7	93,9	**167,9**	129,2	107,3	203,9	192,2
17	**97,5**	198,4	147,2	99	196,2	149,5	98,4	188,3	**137,2**	122,5	**185,8**	218,4
18	109,6	211,1	**154,3**	**107,1**	211,3	161,1	109,1	**200**	157,6	133,8	231,1	233,6
19	115,7	**234,4**	171,6	115,7	235,3	174,5	**112,2**	242,4	**161,3**	147,1	243	248,6
20	188,5	250	186,8	189,3	249	**179,6**	185	245,9	181,2	**162,1**	**239,3**	256,1

**Table 5:** The results obtained from the fuzzy controller.

Floor	GA			DNA			AIS			EA		
	Uppeak	Downpeak	Inter floor	Uppeak	Downpeak	Inter floor	Uppeak	Downpeak	Inter floor	Uppeak	Downpeak	Inter floor
10	−27,5	−26,7	−27,2	−26,1	−22,7	−26,1	**−28,0**	**−27,5**	**−28,0**	−25,8	−26,0	−25,9
11	−24,8	−21,1	−22,7	−24,7	−20,1	−24,7	**−26,6**	−23,4	−24,4	−25,2	**−25,2**	**−25,2**
12	−24,8	−10,5	−21,1	−22,0	−14,2	−22,0	−23,9	−16,5	−20,2	**−25,0**	**−25,0**	**−25,0**
13	−21,4	−3,1	−10,3	−19,5	−6,11	**−19,5**	−20,9	−2,84	−7,30	**−25,1**	**−7,96**	−10,8
14	−18,5	0,25	−6,17	−18,6	**0,03**	**−18,6**	−16,7	2,92	0,35	**−22,1**	15,1	−3,17
15	**−17,5**	9,56	2,31	−16,3	**6,76**	**−16,3**	−12,2	8,09	1,22	−16,5	19,6	17,3
16	−11,7	15,2	6,91	**−16,4**	12,3	**−16,4**	−16,1	**11,3**	2,26	−4,37	24,3	19,8
17	−**6,97**	25,7	8,54	−6,5	22,2	**−6,58**	−6,4	**17,8**	6,64	8,4	20,1	24,5
18	0,33	26,9	12,6	0,6	**24,0**	**0,64**	**−1,1**	26,3	11,6	11,6	25,1	25,0
19	4,9	26,3	16,2	3,3	25,3	**3,30**	**0,7**	25,4	15,5	15,7	**23,1**	22,7
20	22,19	**24,1**	21,4	22,5	25,2	22,5	21,6	25,3	20,3	**18,7**	27,8	**20,1**
